# Rocuronium-sugammadex use for electroconvulsive therapy in a hemodialysis patient: a case report

**DOI:** 10.1186/s40981-016-0055-4

**Published:** 2016-10-10

**Authors:** Shigeaki Kurita, Katsuyuki Moriwaki, Kazuhisa Shiroyama, Mikako Sanuki, Yukari Toyota, Minoru Takebayashi

**Affiliations:** 1 Department of Anesthesiology, Critical Care and Pain Medicine, National Hospital Organization (NHO) Kure Medical Center and Chugoku Cancer Center, Aoyamacho 3-1, Kure-shi, Hiroshima 737-0023 Japan; 2 Department of Psychiatry, National Hospital Organization (NHO) Kure Medical Center and Chugoku Cancer Center, Aoyamacho 3-1, Kure-shi, Hiroshima 737-0023 Japan

**Keywords:** Sugammadex, Rocuronium, Electroconvulsive therapy, Hemodialysis

## Abstract

**Background:**

Recently, rocuronium with subsequent use of sugammadex was proposed for electroconvulsive therapy (ECT) as an alternative to succinylcholine. Because sugammadex is cleared via the kidney with no metabolism, it is unknown that rocuronium-sugammadex use is safe in hemodialysis patients who received ECT.

**Case presentation:**

In this case report, we used rocuronium with subsequent administration of sugammadex in a 69-year-old female, hemodialysis patient, scheduled for ten ECT sessions for severe major depression. In the initial eight sessions, we tested the feasibility of rocuronium-sugammadex use for ECT. During the series of four ECT sessions, we measured plasma concentrations for the sum of sugammadex and sugammadex-rocuronium complex and observed whether possible residual sugammadex affected muscle relaxation during subsequent sessions of ECT. The results showed the feasibility of rocuronium-sugammadex use as muscle relaxants for ECT in patients undergoing hemodialysis. However, an accumulation of sugammadex did occur even after two sessions of hemodialysis, and residual sugammadex decreased the effect of the rocuronium given in the subsequent ECT sessions. Rocuronium-sugammadex was successfully utilized as muscle relaxants for ECT in this patient.

**Conclusions:**

Our experience in this case may indicate that if succinylcholine is contraindicated, rocuronium-sugammadex can be an alternative method for muscle relaxation during ECT in patients undergoing hemodialysis. When this rocuronium-sugammadex procedure is used, the effect of residual sugammadex after hemodialysis on the subsequently administered rocuronium should be considered.

## Background

Depression is common in patients undergoing hemodialysis [1]. Several case reports have indicated that electroconvulsive therapy (ECT) is effective for treatment of depression in hemodialysis patients [2–4]. Although succinylcholine was used in those case reports [2–4], it may cause serious adverse effects such as hyperkalemia and severe bradycardia [5] and is contraindicated in patients with closed-angle glaucoma, susceptibility to malignant hyperthermia, neuroleptic malignant syndrome, and plasma cholinesterase deficiency [5–8]. Recently, rocuronium with subsequent use of sugammadex was proposed for ECT as an alternative to succinylcholine [9]. Sugammadex is cleared almost exclusively via the kidney, with minimal or no metabolism [10]; therefore, it is not recommended for patients with end-stage renal failure and hemodialysis [5, 10]. However, because sugammadex is dialyzable [11], it can be used in hemodialysis patients. In this case report, we tested the feasibility of rocuronium-sugammadex use for ECT in a hemodialysis patient. In addition, we measured plasma concentrations for the sum of sugammadex and sugammadex-rocuronium complex and observed whether possible residual sugammadex affected muscle relaxation during subsequent sessions of ECT.

## Case presentation

A 69-year-old female patient, 133 cm in height and weighing 25 kg, was scheduled for ten ECT sessions for severe major depression. She had a history of chronic renal failure, and hemodialysis was induced 6 months before consultation to the psychiatric department of Kure Medical Center. She had a history of bronchial asthma. Brain MRI showed brain atrophy and old fine cerebral infarctions. She was diagnosed with depression with a mixed type of cognitive impairment. Psychiatric medical therapy was started, but her depressive state worsened, and she was admitted by legal control. After admission, she had hemodialysis three times a week, basically on Monday, Wednesday, and Friday. Every hemodialysis was performed for 3 h using a high-flux membrane (FX140™, Fresenius, Germany). In spite of medical therapy, her symptoms deteriorated. Therefore, ten sessions of ECT were planned after obtaining informed consent from her family. Because of possible succinylcholine-induced hyperkalemia, we initially applied a rocuronium-sugammadex procedure for muscle relaxation of ECT. We obtained informed consent on the following anesthesia protocols from the patient’s family and approval of our IRB (IEC201504). All sessions of ECT were performed in the operating room. Blood pressure, heart rate, and oxygen saturation were monitored using anesthesia monitor (Datex-Ohmeda S/5™, GE Healthcare, Finland). The adductor pollicis train of four (TOF) ratio using kinemyography (NNT MechanoSensor™, GE Healthcare, Finland) was also monitored.

We determined the appropriate doses of rocuronium and sugammadex required for ECT during the initial four sessions. ECT was performed once a week on Wednesday morning, where the patient had hemodialysis in the afternoon, and on Friday and the Monday before the next ECT session. Before induction of anesthesia, surface electrodes were positioned over the ulnar nerve at the wrist. The MechanoSensor™ was attached between the thumb and index finger with a piece of tape. Anesthesia was inducted by propofol with a target propofol concentration in the blood of 2 μg/mL. After automatic calibration, supramaximal TOF stimulation was measured at 20-s intervals. Rocuronium was given with an incremental dose of 5 mg to obtain the TOF ratio of 0 %, and assisted mask ventilation was initiated with 100 % oxygen, followed by a bilateral electrical stimulation of a brief-pulse wave for approximately 5 s (Thymatron System IV™, Somatic Inc., USA). Immediately after seizure stopped, sugammadex was given with an incremental dose of 25 mg for antagonizing rocuronium to achieve recovery of the TOF ratio of 100 %. Hypertension induced by ECT was treated with intravenous nicardipine. We titrated the doses of rocuronium and sugammadex during these sessions. Throughout these four sessions, 20 mg rocuronium and 75 mg sugammadex were found adequate to obtain 0 % and 100 % of the TOF ratios, respectively.

During the fifth to eighth sessions, ECT was given twice a week on Tuesday and Thursday as ordinary procedures [12]. Changes of plasma concentrations for the sum of sugammadex and its rocuronium complex were studied. Fixed doses of 20 mg rocuronium and 75 mg sugammadex were given throughout the sessions. The procedures for induction of anesthesia, muscular blockade, ECT, and reversal of rocuronium were the same as in the initial four sessions. Plasma concentrations for the sum of sugammadex and its rocuronium complex were measured as follows: 5 mL blood samples were obtained immediately before and after hemodialysis via a dialysis needle on the day before the fifth ECT and the next day after the fifth to eighth ECT. Blood samples were centrifuged in 2000–3000*g*, and 2 mL of plasma was cryopreserved at −20 °C. Later, plasma concentrations for the sum of sugammadex and its rocuronium complex were determined using validated liquid chromatographic assay methods with mass spectrometric detection at the Institute of Applied Medicine Inc., Hokkaido, Japan. The lower limit of quantification for the assays was 0.1 μg/mL.

**Fig 1:**
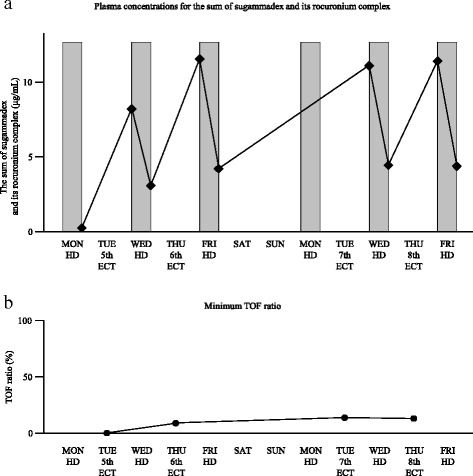
**a** Plasma concentrations for the sum of sugammadex and sugammadex-rocuronium complex. *Gray bars* represent hemodialysis, and *black diamonds* show the concentration before and after hemodialysis. **b** Minimum train of four ratio immediately before electroconvulsive therapy. *HD* hemodialysis, *ECT* electroconvulsive therapy, *TOF* train of four

Changes in plasma concentrations for the sum of sugammadex and its complex are shown in Fig. 1a, and minimum TOF ratio immediately before electroshock is shown in Fig. 1b. Briefly, the sum of sugammadex and its complex concentration was 0.16 μg/mL immediately after three sessions of hemodialysis on the day before the fifth ECT. The values of the plasma concentrations before and after hemodialysis on the next day of the fifth ECT were 8.2 and 3.1 μg/mL, respectively. They were 11.5 and 4.2 μg/mL for the sixth ECT, 11.1 and 4.5 μg/mL for the seventh ECT, and 11.4 and 4.3 μg/mL for the eighth ECT. The changes in concentrations showed that an accumulation of sugammadex and its rocuronium complex did occur. The average reduction ratio (RR) for the sum of the sugammadex and its rocuronium complex by hemodialysis was 61.9 %. The TOF ratios became 0 % after administration of 20 mg rocuronium in the first to fifth ECT, but TOF ratios remained 8–14 % in the sixth to eighth ECT, indicating residual free sugammadex captured rocuronium in part and decreased the effect of rocuronium.

Ten sessions of ECT were performed without complications, and her depressive state dramatically improved. There was no recurarization, muscular weakness, or respiratory problems encountered after ECT during the sessions.

### Discussion

Although not recommended for use in patients with severe renal impairment [5, 10], sugammadex can reverse rocuronium-induced neuromuscular blockade effectively in such patients [13]. Our experience in this case report showed the feasibility of rocuronium-sugammadex use for ECT in patients undergoing hemodialysis. However, an accumulation of sugammadex did occur even after two sessions of hemodialysis (Fig. 1a). Cammu et al. reported that hemodialysis using a high-flux dialysis method was effective in removing sugammadex and its rocuronium complex in patients with severe renal impairment [11]. In their intensive care patients, the observed RR in the plasma concentrations of sugammadex was 69 % during the first dialysis. The value of the RR was similar to the 61.9 % observed as the average RR in the sum of sugammadex and its rocuronium complex in our patient. An RR of 60–70 %, however, may indicate inadequate removal of sugammadex and its rocuronium complex in a single session or two sessions of hemodialysis. In this case report, we confirmed that residual sugammadex decreased the effect of the fixed dose of rocuronium given in the subsequent ECT sessions (Fig. 1b).

The prevention of bone fractures during ECT is especially important in hemodialysis patients because of the prevalence of osteopenia in such patients [14]. A twitch depression of 11–25 % was reported as appropriate in one study, but the optimal level of neuromuscular blockade for ECT remains largely unknown [5]. In our patient, we used a fixed dose of rocuronium and sugammadex during the fifth to eighth ECT sessions and allowed an increase of the TOF ratio up to 14 %. More paralysis might be safer in order to prevent fractures, although no such complication was encountered in our patient.

Although we confirmed that the combination of rocuronium and sugammadex is a feasible alternative muscle relaxation for ECT even in patients with hemodialysis, the high costs of sugammadex may be a challenge economically [15]. The possibility of rocuronium- or sugammadex-induced anaphylaxis [16, 17] and the risk of recurarization [18] are also other concerns in the use of the rocuronium-sugammadex combination. Further study is needed to evaluate the risk- and cost-benefits for use of rocuronium-sugammadex for ECT in hemodialysis patients.

## Conclusions

We successfully used the rocuronium-sugammadex procedure over eight sessions as muscle relaxation for ECT in a hemodialysis patient. If succinylcholine is contraindicated, the rocuronium-sugammadex combination may be an alternative method for muscle relaxation during ECT in patients undergoing hemodialysis. When this alternative procedure is used, neuromuscular monitoring such as quantitative measurement of the TOF ratio should be used to titrate the dose of rocuronium while considering the effect of residual sugammadex after hemodialysis.

## Abbreviations

ECT: Electroconvulsive therapy
RR: Reduction ratio
TOF: Train of four
